# Quadruple space-group ambiguity owing to rotational and translational noncrystallographic symmetry in human liver fructose-1,6-bisphosphatase

**DOI:** 10.1107/S2059798316016715

**Published:** 2016-10-28

**Authors:** Armin Ruf, Tim Tetaz, Brigitte Schott, Catherine Joseph, Markus G. Rudolph

**Affiliations:** a pRED, Therapeutic Modalities, F. Hoffmann-La Roche, 4070 Basel, Switzerland

**Keywords:** pseudo-symmetry, fructose-1,6-bisphosphatase, allosteric regulation, conformational change, glucose metabolism

## Abstract

Fructose-1,6-bisphosphatase (FBPase) is a key regulator of gluconeogenesis and a potential drug target for type 2 diabetes. FBPase is a homotetramer of 222 symmetry with a major and a minor dimer interface. The dimers connected *via* the minor interface can rotate with respect to each other, leading to the inactive T-state and active R-state conformations of FBPase. Here, the first crystal structure of human liver FBPase in the R-state conformation is presented, determined at a resolution of 2.2 Å in a tetragonal setting that exhibits an unusual arrangement of noncrystallographic symmetry (NCS) elements. Self-Patterson function analysis and various intensity statistics revealed the presence of pseudo-translation and the absence of twinning. The space group is *P*4_1_2_1_2, but structure determination was also possible in space groups *P*4_3_2_1_2, *P*4_1_22 and *P*4_3_22. All solutions have the same arrangement of three *C*_2_-symmetric dimers spaced by 1/3 along an NCS axis parallel to the *c* axis located at (1/4, 1/4, *z*), which is therefore invisible in a self-rotation function analysis. The solutions in the four space groups are related to one another and emulate a body-centred lattice. If all NCS elements were crystallographic, the space group would be *I*4_1_22 with a *c* axis three times shorter and a single FBPase subunit in the asymmetric unit. *I*4_1_22 is a minimal, non-isomorphic supergroup of the four primitive tetragonal space groups, explaining the space-group ambiguity for this crystal.

## Abstract 2

The crystal structure of the liver isoform of human fructose-1,6-bisphosphatase in the active R-state conformation was determined by molecular replacement using data from a crystal with noncrystallographic rotational symmetry and pseudo-translation. Owing to an almost perfect placement of noncrystallographic symmetry elements, quadruple space-group ambiguity within the same Laue symmetry arises, including two enantiogenic pairs. The origins of space-group ambiguity, the assignment of the correct space group, refinement and model properties are discussed.

## 1. Introduction   

**Figure 1:**
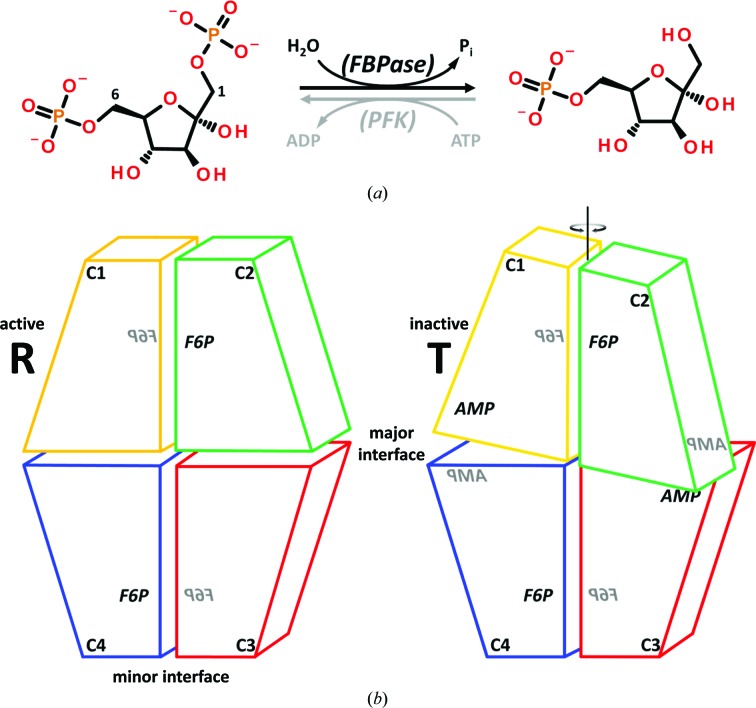
Catalysis and conformational changes of hlFBPase. (*a*) Hydrolysis of fructose 1,6-bisphosphate to fructose 6-phosphate (F6P) by FBPase. The reverse reaction is catalyzed by phosphofructokinase (PFK) at the expense of ATP. (*b*) Schematic view of the FBPase tetramer and the conformational switch from the active R state (no AMP bound) to the inactive T state (AMP bound). The protomers are labelled C1–C4, with C1/C2 and C3/C4 constituting the functional units that rotate with respect to each other. The active sites for hydrolysis are close to the minor interfaces of these dimers (labelled F6P with the dynamic loop nearby) and the allosteric AMP-binding sites are close to the major interfaces (labelled AMP in the right-hand panel).

Glucose is the main energy source for the brain. In mammals, blood glucose homeostasis is maintained mainly by the balance of catabolic glycolysis on the one hand and (with respect to glucose) anabolic glycogenolysis and gluconeogenesis on the other. Increased glucose production is the predominant cause of high blood glucose levels in type 2 diabetes, which can lead to kidney, neurological and cardiovascular damage. In humans, high glucose levels arise from excessive gluconeogenesis in the liver rather than from glycogenolysis of hepatic glycogen stores. Fructose 1,6-bisphosphatase (FBPase) is a major control point in gluconeogenesis, catalyzing the hydrolysis of fructose 1,6-bisphos­phate (F-1,6-P_2_) to fructose 6-phosphate (F6P) and inorganic phosphate (Fig. 1 ▸ *a*). This step in gluconeogenesis is synergistically down-regulated by fructose 2,6-bisphosphate (F-2,6-P_2_) and AMP, which bind to the active site and an allosteric site of FBPase, respectively. While the cellular level of AMP seems to be constant (Xue *et al.*, 1994 ▸), the concentration of F-2,6-P_2_ is controlled by the glucagon-sensitive enzyme 6-phosphofructo-2-kinase/fructose-2,6-bisphosphatase. A small change in the F-2,6-P_2_ concentration thus has a large effect on AMP-mediated FBPase inhibition. During times of glucose demand, F-2,6-P_2_ levels are reduced, leading to increased activity of FBPase. An aberrant up-regulation of gluconeogenesis, especially when coupled with decreased uptake and metabolism of glucose from the blood into cells, may lead to type 2 diabetes (Visinoni *et al.*, 2012 ▸). Inhibition of the liver isoform of FBPase (hlFBPase; the other isoform being the muscle isoform) is therefore an attractive avenue for disease treatment.

FBPases are homotetramers with *D*_2_ symmetry composed of 37 kDa subunits. Each subunit contains one active and one allosteric site (Fig. 1 ▸ *b*). The subunits are labelled C1–C4 and form two functional dimers: C1/C2 and C3/C4. The active site of the C1 subunit is near the C1/C2 interface, while its AMP-binding site is near the C1/C4 interface. The C1/C2 and C3/C4 dimers can rotate with respect to each other. In the enzymatically active R state of the hlFBPase tetramer, the dimers are little, if at all, rotated and the protomers are therefore arranged as an almost planar rectangle. In this conformation, a loop important for catalysis (residues 50–72, also termed the ‘dynamic loop’) may either be disordered or folds onto the active site, allowing F-1,6-P_2_ to be hydrolyzed (Choe *et al.*, 1998 ▸). The hydrolysis reaction requires the presence of Mg^2+^ or Zn^2+^ ions. In contrast, binding of the allosteric regulator AMP facilitates rotation of the dimers by ∼15°, leading to detachment of the catalytic loop (Choe *et al.*, 1998 ▸). This T state of FBPase is the catalytically inactive conformation.

Structural information on FBPases is available for porcine (no isoforms), rabbit, human liver and human muscle FBPases in the T state (Ke, Zhang *et al.*, 1990 ▸; Gidh-Jain *et al.*, 1994 ▸; Iversen *et al.*, 1997 ▸; Barciszewski *et al.*, 2016 ▸) and for porcine and human muscle FBPase in the R state (Ke *et al.*, 1989 ▸; Ke, Zhang *et al.*, 1990 ▸, Ke, Liang *et al.*, 1991 ▸; Ke, Zhang *et al.*, 1991 ▸; Choe *et al.*, 1998 ▸, 2000 ▸; Xue *et al.*, 1994 ▸; Weeks *et al.*, 1999 ▸; Barciszewski *et al.*, 2016 ▸). Crystal structures of porcine FBPase in complex with various ligands established that the R and T states differ from each other by an ∼15° rotation of the dimers about the principal molecular axis (Ke, Zhang *et al.*, 1990 ▸; Choe *et al.*, 1998 ▸). Pushing the FBPase conformational equilibrium towards the inactive T state by allosteric inhibitors that mimic AMP is a well explored route for glucose-level modulation in the blood (Wright *et al.*, 2003 ▸; von Geldern *et al.*, 2006 ▸; Dang *et al.*, 2007 ▸, 2008 ▸, 2011 ▸; Erion *et al.*, 2005 ▸, 2007 ▸; Lai *et al.*, 2006 ▸; Kitas *et al.*, 2010 ▸; Hebeisen *et al.*, 2011 ▸; Tsukada *et al.*, 2009 ▸, 2010 ▸). AMP-site inhibitors connected by a suitable linker to simultaneously bind the AMP sites from two adjacent subunits in the FBPase tetramer gain 10^5^ times in potency compared with monomeric inhibitors (Hebeisen *et al.*, 2008 ▸). In addition, a second allosteric site at the C1/C4 interface that is also close to the C1/C2 interface, and hence common to all subunits, has been exploited for inhibitor design (Wright *et al.*, 2002 ▸; Choe, Nelson *et al.*, 2003 ▸).

For human liver FBPase, currently only T-state structures have been published. Here, we have determined the structure of hlFBPase in a novel primitive tetragonal crystal form in the active R state. An interesting combination of rotational NCS (rNCS) and translational NCS (tNCS) allows structure determination in four different space groups comprising two enantiogenic pairs. The combination of a twofold rNCS at a special position with tNCS of vulgar fractions of a unit-cell length leads to pseudo-body-centred tetragonal symmetry, but the true space group is *P*4_1_2_1_2. Here, we describe how the rNCS and tNCS emulate pseudo-symmetry and discuss the quaternary structure of hlFBPase in light of the R/T transitions in the pig and human FBPase enzymes.

## 2. Materials and methods   

### 2.1. Protein purification, crystallization and data collection   

Human liver FBPase 1 cDNA (NM_000507) was purchased from Origene, cloned into pET-21a *via* the EcoRI/NdeI sites and produced in *Escherichia coli* BL21 (DE3). Cells were suspended in 20 m*M* Tris–HCl pH 7.5, 1 m*M* EDTA, 1 m*M* DTT and disintegrated using a French press. The soluble extract was heated to 65°C for 5 min. The supernatant after centrifugation was applied onto a Macro-Prep High Q column (Bio-Rad) equilibrated in the above buffer. The flowthrough containing FBPase activity was applied onto a Macro-Prep High S column (Bio-Rad) equilibrated with 20 m*M* HEPES–NaOH pH 7.2, 1 m*M* DTT. hlFBPase was eluted with an NaCl gradient and fractions containing active FBPase were pooled. Size-exclusion chromatography on Sephacryl S200 equilibrated in 20 m*M* Tris–HCl pH 7.5, 150 m*M* NaCl, 1 m*M* EDTA, 1 m*M* DTT completed the purification. For optimal activity in enzymatic reactions, hlFBPase was dialyzed against 10 m*M* potassium/sodium phosphate pH 7.4, 2 m*M* MnCl_2_, 5 m*M* MgCl_2_, 2 m*M* ZnCl_2_.

**Figure 2:**
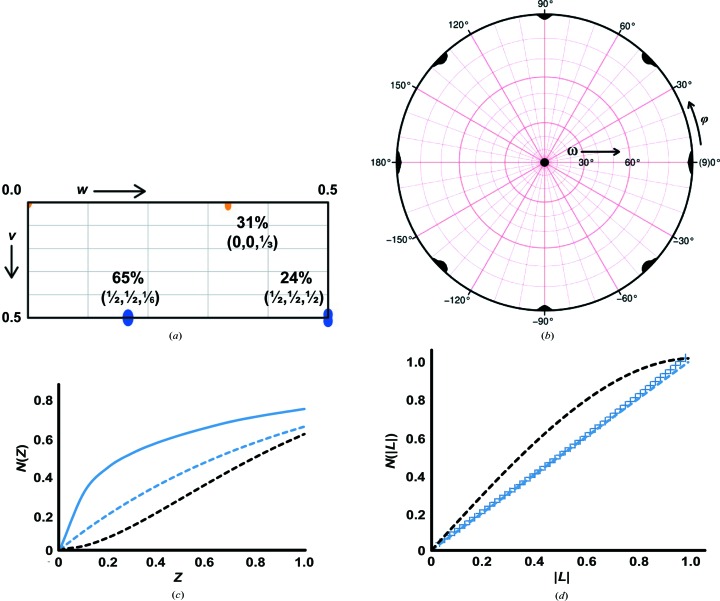
Detection of noncrystallographic symmetry in hlFBPase. (*a*) Superposition of the unique quarters of the *u* = 0 and *u* = 1/2 Patterson sections. The origin peak was removed and only peaks of >10σ are displayed. The *u* = 0 section (orange) contains a single non-origin peak at fractional coordinates (0, 0, 1/3) at a height of 31% of the origin peak. The peaks on the *u* = 1/2 (blue) section at fractional coordinates (1/2, 1/2, 1/6) and (1/2, 1/2, 1/2) have relative heights of 65 and 24%, respectively. Note that the peak at (1/2, 1/2, 1/6) is the sum of the vectors (1/2, 1/2, 1/2) and (0, 0, 1/3). The Patterson function was calculated to 2.5 Å resolution using *XPREP* (Bruker). (*b*) Stereographic projection of the κ = 180° section of the self-rotation function. Peaks occur at the expected positions for 422 symmetry. The function was calculated from data processed in space group *P*422 using *XPREP* (Bruker) with 10–2.5 Å resolution limits and a sphere radius of 55 Å. (*c*) Cumulative intensity distribution *N*(*Z*) of acentric reflections as a function of *Z* = *I*/〈*I*〉. hlFBPase data are drawn as a continuous blue line. Dashed lines represent the theoretical distributions for perfectly twinned (black) and untwinned (blue) data. The shift to larger *N*(*Z*) values indicates pseudo-translation, and possibly masks twinning. (*d*) *L*-test to detect the possible presence of twinning in the presence of pseudo-translation. The cumulative distributions and *N*(|*L*|) of the intensities *I *are plotted as a function of *L* = |*I*_1_ − *I*_2_|/(*I*_1_ + *I*_2_), where *I*_1_ and *I*_2_ are unrelated intensities (Padilla & Yeates, 2003 ▸). hlFBPase data are drawn as a continuous blue line, as in (*c*). Dashed lines are expected distributions for normal (blue) and perfectly twinned (black) data. The observed hlFBPase data show no strong deviations from the untwinned case. The mean value of |*L*| = 0.48 is close to that expected for un-twinned data (Table 1 ▸). The calculations in (*c*) and (*d*) were performed on data scaled in space group *P*4. Plots for data scaled in space group *P*422 look virtually identical.

Crystals were obtained in a micro-batch setup by mixing 0.5 µl volumes of 22.5 mg ml^−1^ hlFBPase in 10 m*M* potassium/sodium phosphate pH 7.4, 2 m*M* MnCl_2_, 5 m*M* MgCl_2_, 2 m*M* ZnCl_2_, 0.5 m*M* F-2,6-P_2_ with reservoir solution consisting of 0.1 M Tris–HCl pH 8.5, 2 *M* ammonium sulfate. Crystals were cryoprotected and a data set was collected to a resolution of 2.2 Å (Table 1 ▸) from a cryocooled single crystal at 100 K on beamline PX-II at the Swiss Light Source using a wavelength of 0.979 Å and a MAR CCD detector of 165 mm diameter. Data were integrated and scaled using the *HKL* package (Otwinowski & Minor, 1997 ▸) and *SADABS* (Bruker), respectively. Indexing and integration was possible in primitive tetragonal, but not *I*-centred. settings, leading to unit-cell parameters of *a* = 121.5, *c* = 316.6 Å (Table 1 ▸). The likely presence of fourfold and twofold screw axes was established by analysis of the systematically absent reflections from data processed in space group *P*422 (Table 2 ▸). As discussed in §[Sec sec2.3]2.3, the true space group is *P*4_1_2_1_2 and the presence of pseudo-translation (Fig. 2 ▸ *a*) emulates *I*-centred symmetry. Assuming six FBPase molecules in the asymmetric unit, the Matthews coefficient (Matthews, 1968 ▸) is 2.8 Å^3^ Da^−1^ with a solvent content of 56%.

### 2.2. Data analysis   

The data indexed readily and produced reasonable scaling statistics in a primitive 422 lattice (Table 1 ▸). To establish the space group, systematic absences were analyzed. While no reflections were measured along the *h* axis, analysis of the 0*k*0 reflections is consistent with the presence of a 2_1_ screw axis. Likewise, the presence of only *l* = 4*n* reflections is consistent with both a 4_1_ and a 4_3_ screw axis, suggesting *P*4_1_2_1_2 or its enantiomorph as possible space groups (Table 2 ▸). Plots of the self-rotation function calculated with *XPREP* (Bruker) in space group *P*422 are unremarkable, displaying the expected fourfold (κ = 90°; not shown) and the mutually perpendicular arrangement of the fourfold and twofold axes in the κ = 180° section (Fig. 2 ▸ *b*). Should noncrystallographic rotational symmetry be present, these axes would have to run parallel to a crystallographic axis. The *u* = 0 and *u* = 1/2 sections of the native Patterson map (Fig. 2 ▸ *a*) contain three significant peaks of >20% of the origin signal at positions (*u*, *v*, *w*) = (1/2, 1/2, 1/6), (0, 0, 1/3) and (1/2, 1/2, 1/2). The presence of strong pseudo-translation manifests itself in a high value for the standard deviation of the mean normalized structure-factor amplitude *E*, 〈|*E* ^2^ − 1|〉, which is 1.152 but should be 0.736 for chiral space groups in the absence of twinning (Table 1 ▸), and also in the cumulative intensity distribution as reported by *TRUNCATE* from *CCP*4 (Winn *et al.*, 2011 ▸; Fig. 2 ▸ *c*). If the special position at (1/2, 1/2, 1/6) were crystallographic, no intensities would be measurable for a subset of reflections, while in the case of noncrystallographic symmetry certain reflections would be weak. Querying the structure-factor formula for reflection conditions that result in amplitudes |*F*_*hkl*_| = 0 requires
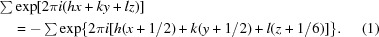
This leads to the condition exp[π*i*(*h* + *k* + *l*/3)] = −1, which is fulfilled by 1/6 of all reflections in the data set that obey *h* + *k* + *l*/3 = 2*n* + 1, with *n* being an integer. While these ∼20 000 reflections are indeed weak, they are not absent, but have an 〈*I*〉/〈σ(*I*)〉 value of 2.0 (Table 2 ▸). For comparison, the *h* + *k* + *l*/3 = 2*n* reflections have correspondingly larger intensities of 〈*I*〉/〈σ(*I*)〉 = 19.6, while 〈*I*〉/〈σ(*I*)〉 for the whole data set is 11.8 (Table 2 ▸). An equivalent reasoning for the weaker Patterson peak at (1/2, 1/2, 1/2) reveals only a slight decrease of 18% in the intensities of reflections *h* + *k* + *l* = 2*n* + 1 (Table 2 ▸) compared with the *h* + *k* + *l* = 2*n* reflections, in line with the impossibility of indexing the data in an *I*-centred lattice where reflections *h* + *k* + *l* = 2*n* + 1 must be absent. Lastly, the vector (0, 0, 1/3) will not change any reflection intensities. The source of the pseudo-translation and pseudo-centring only became clear after molecular replacement (discussed below). Owing to the presence of pseudo-translation and the corresponding strong correlation of some reflection intensities, global statistics that rely on the data following a Wilson distribution are skewed (Table 1 ▸). Thus, twinning tests based on the cumulative intensity distribution, the |*E* ^2^ − 1| value (Fig. 2 ▸ *c*) or moments on intensities or structure-factor amplitudes cannot be employed, and the pseudo-translation may mask the presence of twinning (Padilla & Yeates, 2003 ▸; Rudolph *et al.*, 2004 ▸; Zwart, Grosse-Kunstleve *et al.*, 2008 ▸; Read *et al.*, 2013 ▸). It is possible in principle that the hlFBPase data belong to Patterson symmetry *P*4/*m* with twinning emulating the higher metric symmetry *P*4/*mmm*. A twinning test based on local non-twin-related reflection pairs, the *L*-value (Padilla & Yeates, 2003 ▸), which is not influenced by the presence of pseudo-centring provided that suitable reflection pairs are chosen (Sliwiak *et al.*, 2015 ▸), shows little deviation from the expected values for untwinned data (Table 1 ▸, Fig. 2 ▸ *d*), indicating that the hlFBPase data are not twinned. This turned out to be true after refinement (see below).

### 2.3. Phasing and refinement   

Molecular replacement was performed to phase the hlFBPase data in the four space groups *P*4_1_22, *P*4_3_22, *P*4_1_2_1_2 and *P*4_3_2_1_2 using *Phaser* v.2.1.4 (McCoy *et al.*, 2007 ▸). FBPase is a homotetramer composed of a dimer of dimers. The C1/C2 dimer of an in-house FBPase structure was used as the search model. When tested separately, all space groups returned a single solution of three dimers with the same overall arrangement, whereby space group *P*4_1_2_1_2 gave the strongest signal (Table 3 ▸). Of note, the latest version of *Phaser* will use the tNCS information present in the data to automatically select the correct space group. Refinement of all four solutions in *BUSTER* (Blanc *et al.*, 2004 ▸) using the same set of test reflections to calculate *R*_free_ indicated *P*4_1_2_1_2 as the correct space group based on the lowest *R*_free_ value (Table 3 ▸). The model in space group *P*4_1_2_1_2 was rebuilt in *Coot* (Emsley *et al.*, 2010 ▸) and refined using *BUSTER*. No NCS restraints were applied and each of the six protomers was defined as a TLS group. Loop 22–26 near the AMP-binding site and the catalytic loop 56–70 were not included in the model owing to a lack of electron density. Refinement statistics are collected in Table 4 ▸. Coordinates and structure factors for hlFBPase have been deposited in the Protein Data Bank (PDB entry 5ldz). Analysis of electron-density maps calculated with anomalous differences and refined phases revealed significant peaks of >5σ that could be either Zn^2+^ or Mn^2+^. At the wavelength of data collection (0.979 Å), the calculated values for zinc and manganese are *f*′′(Zn) = 2.5 e and *f*′′(Mn) = 1.3 e, pointing to zinc rather than manganese as the element present. As discussed in §3.4, owing to the 3.3-fold more frequent occurrence of trigonal bipyramidal coordination of zinc *versus* manganese in the Cambridge Structural Database (CSD) and previous FBPase crystal structures containing Zn^2+^ rather than Mn^2+^, the former ion was modelled at these positions (Fig. 8), although a mixture of Zn^2+^ and Mn^2+^ cannot be completely excluded.

## 3. Results and discussion   

### 3.1. Combination of rNCS and tNCS in FBPase   

**Figure 3:**
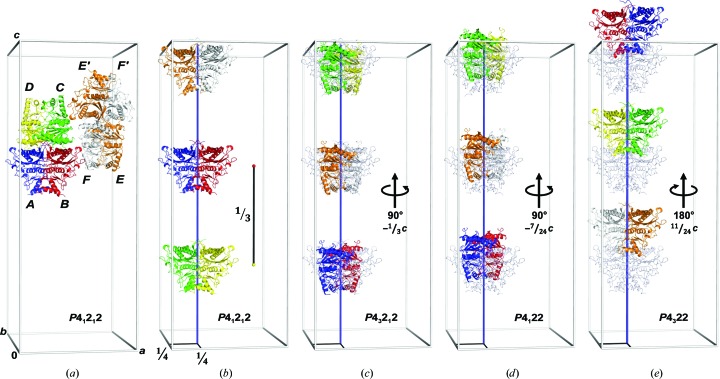
Molecular-replacement solutions for the hlFBPase crystal. (*a*) The six protomers (*A*–*F*) in the asymmetric unit of space group *P*4_1_2_1_2, shown as differently coloured ribbons, form three dimers. Two of the C1/C2 dimers (*AB* and *CD*) associate to form a homotetramer, the biological unit of FBPase. The third dimer (*EF*) is complemented to form a tetramer by crystal symmetry (*E*′*F*′). (*b*) A different choice of the asymmetric unit visualizes a pseudo-translation vector (black bar) of fractional coordinates (0, 0, 1/3), parallel to the *c* axis of the unit cell. The pseudo-translation vector relates dimers *CD*/*AB*, *AB*/*EF* and *EF*/*CD*. The dimers are placed along a twofold NCS axis at (1/4, 1/4, *z*) that is drawn in blue. (*c*, *d*, *e*) Molecular-replacement solutions for space groups *P*4_3_2_1_2, *P*4_1_22 and *P*4_3_22 in the same colour code as in (*a*), with the *P*4_1_2_1_2 solution shown in grey as a reference. The search model was the *P*4_1_2_1_2 solution. The transformation from the solution in *P*4_1_2_1_2 to the other space group is given.

The three dimers in the asymmetric unit of hlFBPase form one and a half FBPase tetramers, with the first tetramer composed of protomers *A*, *B*, *C* and *D*, and the second (*EF* *E*′*F*′) completed by crystal symmetry (Fig. 3 ▸ *a*). Alignment of molecular symmetry axes with crystallographic elements is quite common for FBPases from different organisms. 30 out of 90 structures in the PDB, including pig, rabbit and human FBPases, crystallized in five different space groups and contain only a single subunit in the asymmetric unit. The tetramer is constructed by crystallographic symmetry operations (Choe *et al.*, 1998 ▸, 2000 ▸; Weeks *et al.*, 1999 ▸; Choe, Iancu *et al.*, 2003 ▸; Choe, Nelson *et al.*, 2003 ▸; Iancu *et al.*, 2005 ▸; Shi *et al.*, 2013 ▸; Gao *et al.*, 2013 ▸; Barciszewski *et al.*, 2016 ▸). 50 more FBPase structures in three different space groups have a C1/C2 dimer in the asymmetric unit, and the tetramer is also completed by crystallographic symmetry. Others, such as pig FBPase in space group *P*2_1_2_1_2_1_, contain a complete tetramer with the shortest molecular twofold axis almost aligned with the *b* axis (Ke, Liang *et al.*, 1991 ▸). The case of hlFBPase is a combination of these examples. One complete tetramer is aligned parallel to the *c* axis. An additional C1/C2 dimer is also aligned with the *c* axis and completed to a tetramer by crystallographic symmetry (Fig. 3 ▸ *a*).

Rearrangement of the three hlFBPase C1/C2 dimers reveals a twofold rNCS axis located at fractional coordinates (1/4, 1/4, *z*) with the dimers spaced by 1/3 along *z* (Fig. 3 ▸ *b*). This twofold axis runs parallel to the crystallographic fourfold, explaining the unremarkable self-rotation function (Fig. 2 ▸ *b*). Since a twofold axis at (1/4, 1/4, *z*) is equivalent to a symmetry operation (−*x* + 1/2, −*y* + 1/2, *z*), combination of this NCS operator with those of the primitive tetragonal space groups of Patterson symmetry *P*4/*mmm* generates eight instances of a (1/2, 1/2, 1/2) translation. The pseudo-body-centring introduced thereby leads to the observed Patterson vector at (1/2, 1/2, 1/2). However, the height of this vector is only 24% of the origin peak (Fig. 2 ▸ *a*), ruling out an *I*-centred lattice. Calculation of the native Patterson map at different high-resolution limits shows that this peak reaches 80% of the origin peak at 12 Å, further showing that the *I*-centring is pseudo. The vector at (1/2, 1/2, 1/2) combines with the pseudo-translation of (0, 0, 1/3) along the NCS axis to form a strong signal at (1/2, 1/2, 5/6), which is symmetry-related to (1/2, 1/2, 1/6), thus explaining all of the peaks in the native Patterson map (Fig. 2 ▸ *a*).

In the presence of tNCS, an additional twinning test is sometimes possible (Rudolph *et al.*, 2004 ▸). As a Patterson map of a twinned crystal contains the atomic distance information of both twin domains but no cross-peaks between them, a pseudo-translation vector in a merohedral twin should appear twice in the native Patterson map. The prerequisite is that the tNCS vectors are not superimposed by the twin operator. For diffraction data scaled in space group *P*4, the potential twin operator emulating pseudo-*P*422 symmetry (*k*, *h*, −*l*) corresponds to a twofold rotational axis bisecting the *ab* plane. Unfortunately, since the pseudo-translation vector in hlFBPase is parallel to the rNCS along the *c* axis, the twin operator would superimpose the tNCS vectors of the twin domains (with the inverse direction), thus not leading to any change in the native Patterson map. Therefore, the potential presence of twinning cannot be tested by analyzing the native Patterson maps in this particular case, and prior to refinement and judging the results based on *R*_free_ (see below), the *L*-test (Padilla & Yeates, 2003 ▸) was the sole statistic on which the absence of twinning in the hlFBPase data was based (Fig. 2 ▸ *c*).

### 3.2. The same packing of hlFBPase in four space groups   

Molecular replacement in the four space groups *P*4_1_22, *P*4_3_22, *P*4_1_2_1_2 and *P*4_3_2_1_2 each resulted in a single unique solution with a log-likelihood gain (LLG) of >16 000 (Table 3 ▸). The solution in *P*4_1_2_1_2 can be transformed into the *P*4_3_2_1_2 solution by a 90° clockwise rotation about (1/4, 1/4, *z*) and a shift of −1/3 along *z* (Fig. 3 ▸c). The corresponding transformation into space group *P*4_1_22 is the same rotation but with a translation by −7/24 along *z* (Fig. 3 ▸ *d*), and the transformation into space group *P*4_3_22 requires a 180° rotation about (1/4, 1/4, *z*) and a shift of 11/24 along *z* (Fig. 3 ▸ *e*). Refinement of hlFBPase in the four space groups led to acceptable *R*_free_ values in all cases, although with a preference for space groups *P*4_1_2_1_2 and *P*4_1_22 (Table 3 ▸). The large cross-correlation coefficients between the phases in all four space groups of 0.81–0.84 confirm the notion that all solutions essentially describe the same situation. The question arises how packing in two enantiogenic space-group pairs is possible or, in other words, how the hlFBPase asymmetric unit can introduce a twofold axis and thereby generate additional fourfold screw axes of both hands (Fig. 3 ▸).

**Figure 4:**
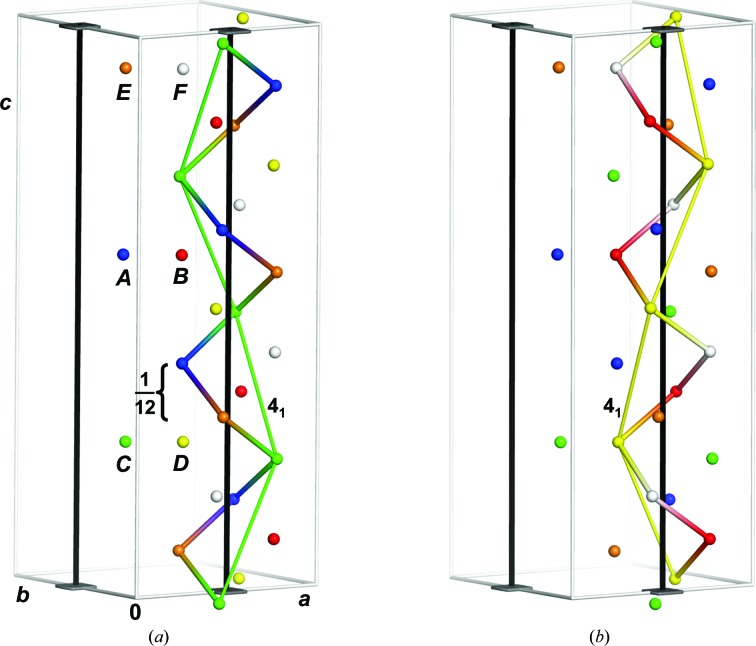
Space-group ambiguity owing to rNCS at (1/4, 1/4, *z*). The six hlFBPase protomers are represented by spheres at the coordinates of the centre of mass. They are coloured individually and labelled *A*–*F*. (*a*) One half of the molecules related by the fourfold axis (black rod) forms a left-handed fourfold screw axis of three complete turns along the *c* axis. Successive molecules are translated by *c*/12. A subset of these molecules is related by a (right-handed) 4_1_ axis (green) connecting symmetry mates. In fact, all like-coloured molecules are related by a 4_1_ axis. (*b*) The other half of the molecules related by the fourfold axis also forms a left-handed helix, which incorporates a 4_1_ axis (yellow), connecting two different symmetry mates in an alternating pattern.

**Figure 5:**
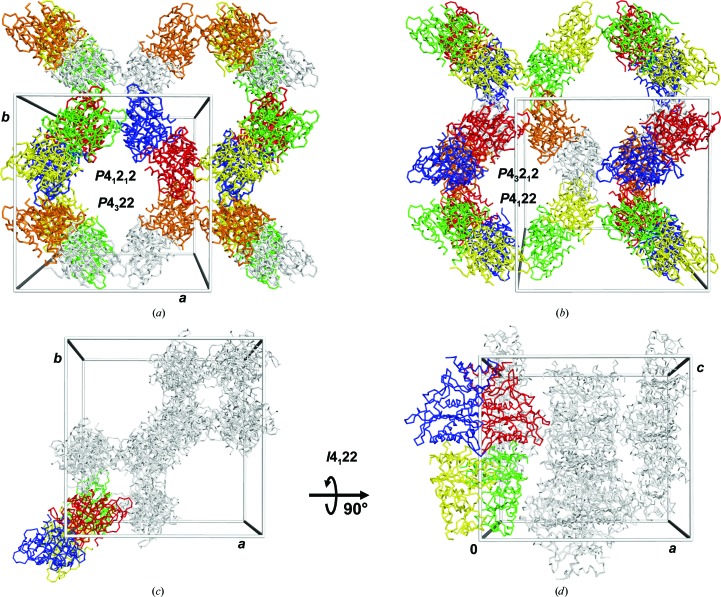
The same packing of hlFBPase in tetragonal space groups. (*a*) Packing arrangement viewed along the *c* axis for the molecular-replacement solutions in space groups *P*4_1_2_1_2 and *P*4_3_22. (*b*) The same view for space groups *P*4_3_2_1_2 and *P*4_1_22. (*c*) Packing of the forced *I*4_1_22 setting with a *c*/3 axis and a crystallographic twofold instead of the observed rNCS. The default setting in the *I*-centred space group has the origin on the twofold axis along the *c* axis, leading to the shift compared with (*a*) and (*b*), but otherwise the packing is virtually identical. (*d*) A view rotated 90° compared with (*c*) shows a complete FBPase tetramer generated by crystal symmetry from the asymmetric unit, which in *I*4_1_22 would be a single subunit.

The handedness of the fourfold screw axis is a distinguishing feature of the enantiomorphic pairs *P*4_1_2_1_2/*P*4_3_2_1_2 and *P*4_1_22/*P*4_3_22. Thus, the arrangement of protomers in the asymmetric unit must enable both hands. Fig. 4 ▸ shows the molecular-replacement solution for *P*4_1_2_1_2 with the six individually coloured protomers simplified by the position of their centres of mass. Half of the molecules around a fourfold axis at (1/2, 0, *z*) follow a left-handed ‘4_1/3_’ axis, *i.e.* a fourfold screw axis with three complete turns along the unit cell: each molecule is shifted by 1/12 with respect to its predecessor (Fig. 4 ▸ *a*). A subset of molecules within this group follows a canonic 4_1_ axis. Likewise, the other half of the molecules around the crystallo­graphic fourfold screw axis also follow a left-handed ‘4_1/3_’ axis, with a subset following a canonic 4_1_ axis (Fig. 4 ▸ *b*). Thus, both types of handedness are present in both subsets, explaining the ambiguity along the fourfold axis. Similar considerations hold for the twofold or 2_1_ axis parallel to the crystallographic *a* (and *b*) axis in the pairs *P*4_1_2_1_2/*P*4_1_22 and *P*4_3_2_1_2/*P*4_3_22. Accordingly, the four molecular-replacement solutions display virtually identical packing (Figs. 5 ▸ *a* and 5 ▸ *b*).

### 3.3. Requirements for and breakdown of the *I*-centring in hlFBPase   

The presence of a twofold axis at (1/4, 1/4, *z*) is a distinguishing feature of the *I*4_1_22 lattice compared with space groups with *P*4/*mmm* Patterson symmetry. In hlFBPase, a twofold rNCS axis is present at this position that would in principle allow *I*4_1_22 as a higher symmetry space group. *I*4_1_22 is a minimal non-isomorphic supergroup for the four space groups *P*4_1_22, *P*4_3_22, *P*4_1_2_1_2 and *P*4_3_2_1_2, further corroborating why molecular replacement was possible in all of them. A 2_1_ screw axis at (1/4, 1/4, *z*) would generate an *I*422 lattice, but the corresponding primitive space groups would be *P*422/*P*42_1_2 or *P*4_2_22/*P*4_2_2_1_2, which are not supported by an analysis of systematic absences (Table 2 ▸), and which also did not deliver molecular-replacement solutions. Reflections with *l* ≠ 4*n* are absent, identifying a 4_1_/4_3_ screw axis in the hlFBPase data, which leaves *I*4_1_22 as the sole option. A caveat for interpreting systematic absences is the possibility of pseudo-translation changing reflection intensities along the reciprocal axes, which might apply to hlFBPase owing to the translational component of ∼1/3 along the *c* axis. As mentioned above, the subset that is strongly altered by the vector (1/2, 1/2, 1/6) is (*h* + *k* + *l*/3), and (0, 0, *l*) reflections with reflection condition *l* = 4*n* for 4_1_ or 4_3_ axes that are a part of this subset must have *l* = 12*n*. Because these reflections are even, they are not eliminated by the pseudo-translation but are slightly stronger [〈*I*〉/〈σ(*I*)〉 = 10, *N* = 11]. An alternative approach is to calculate the reflection condition for absences owing to the (0, 0, 1/3) vector for reflections along the *l* axis. The resulting subset (0, 0, 3*n* + 3/2) is impossible, *i.e.* this pseudo-translation vector does not alter any (0, 0, *l*) reflection intensities. Taken together, the systematic absence analysis for the possible higher symmetry space group is unaffected by the pseudo-translation. Thus, if the rNCS at (1/4, 1/4, *z*) were crystallographic, space group *I*4_1_22 would result. The question remains as to why hlFBPase does not form an *I*-centred lattice.

**Figure 6:**
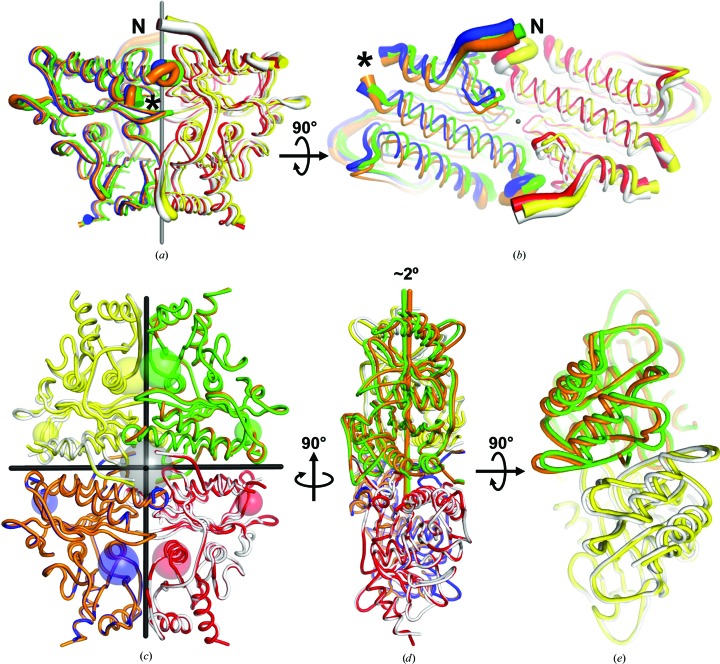
Structural variation of hlFBPase protomers as the origin of pseudo-symmetry. (*a*) Side-on view of the FBPase dimers shifted along the rNCS axis shown in Fig. 3 ▸(*b*). The colour code also follows that in Fig. 3 ▸(*b*). The diameter of the backbone trace corresponds to the mean *B* value of this region, indicating possible flexibility at the N-terminus (labelled) and surface-loop regions. The asterisk denotes a missing loop region (residues 22–26). (*b*) View rotated 90° compared with that in (*a*), showing the lateral displacements of the dimers perpendicular to the rNCS axis in the *ab* plane. (*c*) Superposition of the two hlFBPase tetramers onto one protomer (blue and orange) shows little differences in this orientation. The twofold axes of the almost *D*_2_-symmetric tetramers are marked in black. Important sites are shown as transparent spheres. Small spheres, allosteric AMP-binding sites; grey central sphere, second allosteric site; other large spheres, active and fructose 2,6-bisphosphate-binding sites. (*d*) A rotation by 90° compared with (*c*) reveals a tilted arrangement of dimers. The rotation axes for the upper dimers are drawn as rods, coloured according to one of the protomers, and differ in direction by 2.2°. (*e*) Top view along the rNCS axes shows displacements of the protomers in excess of 2 Å.

The two conditions that need to be fulfilled for *I*-centring are a crystallographic twofold at (1/4, 1/4, *z*) and a pure translation of (0, 0, 1/3). In a case where the translation were exactly 1/3, the three copies of the hlFBPase dimer would be identical and the c* axis would triple (the original *c* axis reduced to 105.5 Å). Space group *I*4_1_22 with a smaller *c* axis would harbour only a single protomer in the asymmetric unit, and crystal symmetry would construct an hlFBPase homotetramer with perfect 222 symmetry, similar to what has been observed with many other FBPase structures (see above). Molecular replacement using data forcibly reduced in space group *I*4_1_22 with a *c*/3 axis resulted in a clear and single solution (*Z*-scores of 11.9 and 27.1 for the rotation and translation functions; overall LLG = 830) with the expected packing (Figs. 5 ▸ *c* and 5 ▸ *d*). However, this solution could not be refined to an *R*_free_ value of <34%, further corroborating the primitive setting. Table 5 ▸ summarizes the geometric relations between the six protomers in the hlFBPase *P*4_1_2_1_2 asymmetric unit. The r.m.s.d. values between the protomers are of the order of 0.3 Å, mostly because of differences in the conformation of the C-termini and the loop region 142–148. The rotation angles are close to either zero or 180°. Nine out of 15 possible twofold rotation combinations vary between 179.0 and 180.0°, with a mean of 179.6 ± 0.3°. The remaining six cases have rotation components between 0.84 and 1.84°, with a mean of 1.21 ± 0.47°. The translation components along the *c* axis are almost exactly 1/3 or 2/3 in all cases. By contrast, the translation vectors in the *ab* plane vary by as much as 4.6% (5.6 Å) from zero or 1/2, indicating that the reason for the breakdown of symmetry, which prohibits an *I*-centred lattice, is owing to lateral translation of the hlFBPase dimers in the *ab* plane. A projection of the FBPase dimers along the rNCS axis onto the *ab* plane confirms this hypothesis: lateral displacements from the rNCS axis are incompatible with this operator being crystallographic (Figs. 6 ▸ *a* and 6 ▸ *b*). In addition, while a superposition of the two hlFBPase tetramers onto one protomer (blue and orange in Fig. 6 ▸ *c*) shows little differences in the face-on orientation (Fig. 6 ▸ *c*), a kinked arrangement of dimers is visible when the tetramers are viewed side-on (Fig. 6 ▸ *d*) or from the top (Fig. 6 ▸ *e*). The directions of the rotation axes for the upper dimers differ significantly by 2.2°, leading to displacements in excess of 2 Å, which is sufficient for the breakdown of body-centred symmetry.

### 3.4. Apo hlFBPase adopts the R state   

**Figure 7:**
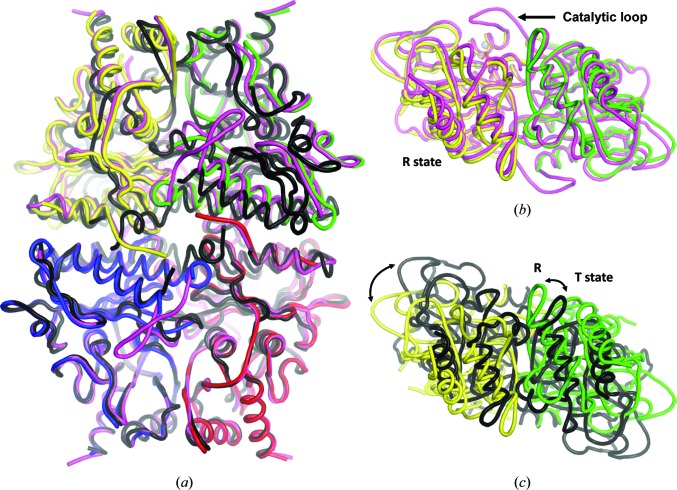
hlFBPase structure and comparison with related FBPases in the R and T states. (*a*) Superposition of hlFBPase (subunits coloured yellow, green, red and blue) with R-state porcine liver FBPase in complex with F6P (Choe *et al.*, 1998 ▸; magenta; PDB entry 1cnq) and with human liver FBPase in the T state (Hebeisen *et al.*, 2011 ▸; black; PDB entry 2y5k). (*b*) View rotated 90° about the horizontal axis to show the close superposition of the R state of apo hlFBPase with the F6P-bound R state of porcine FBPase. (*c*) The same view as in (*b*) but comparing the R state of hlFBPase (green/yellow) with the T state of hlFBPase (black). The two states differ by an ∼15° rotation about the C1/C2 axis.

The crystal structure of hlFBPase described here is the first example of the R state of the human liver isoform (Fig. 7 ▸). The rotation angle of the C1/C2 *versus* the C3/C4 dimers about the principal molecular axis is 2.9°, which is at the upper end of R-state angles of 1.3 ± 0.9° (*n* = 36, range 0–3.8°) found in porcine FBPase crystal structures. A significant deviation from co-planarity (2.8, 3.0 and 5.4°) of the subunits has also been observed for three human muscle FBPase (hmFBPase) structures (Shi *et al.*, 2013 ▸).

Binding of AMP to the allosteric sites induces a conformational change in FBPase from its active R state to its inactive T state (Ke, Thorpe *et al.*, 1990 ▸; Choe *et al.*, 1998 ▸). In human liver FBPases the average rotation angle of the T state is 14.5 ± 0.4° (*n* = 15, range 13.9–15.1°), which is very similar to that observed in porcine FBPase T-state structures of 14.2 ± 1.2 (*n* = 30, range 10.5–17.1°). Three hmFBPase structures have similar rotation angles of 15.6 ± 0.2° (range 15.4–15.7°; Zarzycki *et al.*, 2011 ▸; Barciszewski *et al.*, 2016 ▸), which would indicate that the amount of rotation in FBPases is generally of the order of 15°. This view has been challenged by a recent hmFBPase structure that showed a cruciform arrangement of the dimers, *i.e.* a rotation angle of close to 90° (Barciszewski *et al.*, 2016 ▸). Since the human muscle and liver FBPase isoforms share only 76.9% sequence identity (89.3% homology) over 337 residues and the muscle isoform has additional functions in the cell, including higher sensitivity of hmFBPase to AMP and its regulation by Ca^2+^ (Gizak *et al.*, 2012 ▸; Pirog *et al.*, 2014 ▸), some differences in their R/T transition behaviour might be expected. In solution, porcine FBPase sub­units have been shown to exchange on a time scale of a few hours (Nelson *et al.*, 2001 ▸), indicating flexibility at the interfaces. Thus, the possibly that the rotation angle of FBPases might sometimes be influenced by crystal-packing effects cannot be discounted entirely.

**Figure 8:**
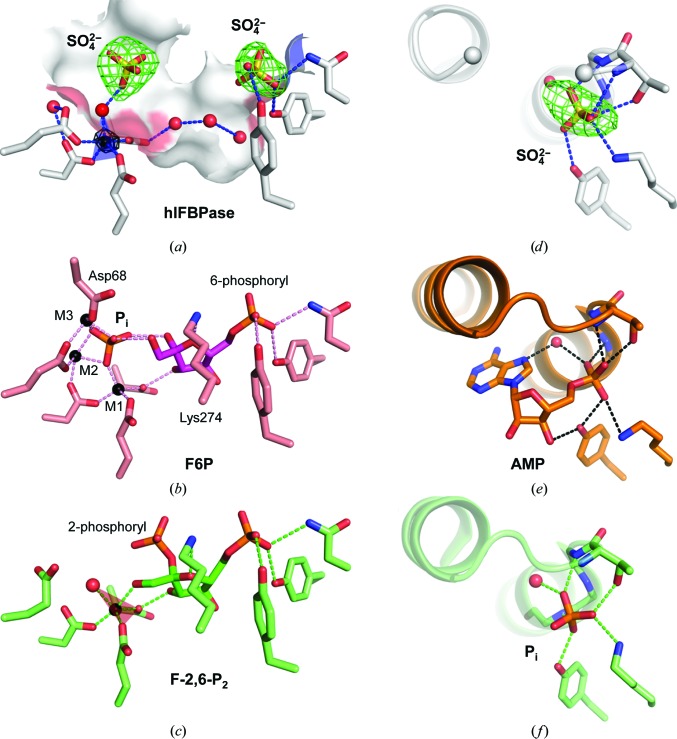
Comparison of the active and allosteric sites. (*a*) The active site in protomer *B* of hlFBPase is shown as a surface. The σ_A_-weighted OMIT electron-density map contoured at 4 r.m.s.d. of the sulfate ions is drawn as a green mesh. The black mesh is a Fourier map using anomalous differences and refined phases as coefficients contoured at 7 r.m.s.d. The Zn^2+^ ion (black sphere) is coordinated by four acidic side chains and a water molecule in trigonal bipyramidal geometry. The transparent blue plane shows the base of the bipyramid. Possible hydrogen bonds (<3.2 Å) are shown as blue dashed lines. In the absence of a carbohydrate, a chain of water molecules (red spheres) fills the active site. (*b*) Active site of porcine FBPase in complex with F6P and phosphate (PDB entry 1cnq). The location of the sufate ions in hlFBPase is close, but not identical, to the phosphates in this product complex. Three Zn^2+^ ions (small black spheres) were found in this structure. In general, the metal sites are denoted M1–M3. A notable difference from the hlFBPase structure is a Lys274 side chain that binds to the O4′ atom of F6P but is flipped away in the apo state of hlFBPase. Asp68 is contributed from the catalytic loop, which has closed onto the active site of FBPase, but is disordered in hlFBPase. (*c*) Active site of porcine FBPase in complex with the competitive inhibitor F-2,6-P_2_ (PDB entry 2qvv). The 2′-phosphoryl group is located halfway between the positions of the inorganic phosphate in the product complex (*b*) and the sulfate ion in apo hlFBPase (*a*). The coordination of the Zn^2+^ ion is trigonal bipyramidal, but in a different arrangement compared with hlFBPase. The transparent blue plane shows the base of the bipyramid. (*d*) Allosteric AMP site in hlFBPase. A loop region is disordered and marked by grey spheres. The red mesh is the σ_A_-weighted OMIT electron-density map contoured at 4 r.m.s.d. around the sulfate ion, which is bound to the phosphate-binding loop (P-loop) and the N-terminus of an α-helix. (*e*) The allosteric AMP site in porcine FBPase in complex with AMP. The loop that is missing in the hlFBPase structure is ordered in this complex. The sulfate ion in hlFBPase binds at the same position as the phosphoryl moiety of AMP. (*f*) Allosteric AMP site in porcine FBPase in complex with sulfate (PDB entry 2qvv). The hydrogen-bonding pattern is the same as in hlFBPase shown in (*d*).

Although excess F-2,6-P_2_ was present during crystallization of hlFBPase to facilitate formation of the R state, the electron density does not support the presence of this competitive inhibitor in the active site. Instead, two sulfate ions from the crystallization medium are located in the active site (Fig. 8 ▸ *a*). Comparison of hlFBPase with porcine FBPase in complex with F6P and phosphate (Choe *et al.*, 1998 ▸) and with porcine FBPase in complex with F-2,6-P_2_ (Hines *et al.*, 2007 ▸) shows that the sulfate ions in hlFBPase are located near the positions of the phosphoryl groups in F6P and F-2,6-P_2_ (Figs. 8 ▸ *b* and 8 ▸ *c*, respectively). The sulfate ion mimicking the 6-phosphoryl group is bound in the same manner in all structures by three hydrogen bonds from side chains of two tyrosines and an asparagine. The second sulfate ion in hlFBPase is located halfway between the positions of the inorganic phosphate in the F6P/P_i_ complex (Fig. 8 ▸ *b*; ∼2.7 Å P–P distance) and the 2-phosphoryl group in the F-2,6-P_2_ complex (Fig. 8 ▸ *c*; ∼2.2 Å P–P distance). While for steric reasons the 2′-phosphoryl group in F-2,6-P_2_ cannot bind to the metal ion, in a total of 11 product complexes the inorganic phosphate is a direct ligand for either Zn^2+^ or Mg^2+^. By contrast, the corresponding sulfate ion in hlFBPase is bound to the metal ion *via* a water molecule, a constellation that has not been observed before in FBPase crystal structures and may reflect a state during catalysis where inorganic phosphate is about to leave the active site after hydrolysis has completed.

Up to three metal ions have been observed in FBPase structures (termed M1–M3; Fig. 8 ▸ *b*). The structure of hlFBPase contains a single metal ion at position M1, which based on the crystallization conditions could in principle be Mg^2+^, Mn^2+^ or Zn^2+^. The M1 site in FBPase seems to be rather promiscuous: while Zn^2+^ was identified in the majority of structures, Mn^2+^, Mg^2+^ and Tl^+^ have also been located at this position. Based on strong OMIT electron density and an anomalous signal of >7 r.m.s.d. (Fig. 8 ▸ *a*), Mg^2+^ could be excluded. Mn^2+^ appears to be less likely than Zn^2+^, since it has only half the anomalous signal at the data-collection wavelength (see §[Sec sec2]2). Also, refinement of hlFBPase with Mn^2+^ at position M1 led to a smaller average *B* value for Mn^2+^ over the six molecules in the asymmetric unit compared with the metal-binding atoms (41 Å^2^ *versus* 49 Å^2^), while refinement with Zn^2+^ yielded average values of 50 Å^2^ for both sets of atoms. A distinction based on distances, which are similar for Zn^2+^ and Mn^2+^ (Harding *et al.*, 2010 ▸), was not possible at the resolution of hlFBPase (2.2 Å). The coordination geometries observed in FBPase crystal structures at the M1 site include tetrahedral, octahedral and trigonal bipyramidal. In the majority of cases the M1 site is occupied by a tetrahedrally coordinated Zn^2+^, reflecting the preferred geometry of this ion. By contrast, the Zn^2+^ in hlFBPase has trigonal bipyramidal coordination from four acidic side chains and a water molecule (Fig. 8 ▸ *a*). A similar trigonal bipyramidal coordination of Zn^2+^, although tilted with respect to hlFBPase, was observed in the crystal structure of porcine FBPase in complex with F-2,6-P_2_ (Hines *et al.*, 2007 ▸; Fig. 8 ▸ *c*). A search in the CSD for zinc- and mangan­ese-containing compounds revealed only 103 small molecules with an Mn atom in trigonal bipyramidal geometry (0.57%), while a Zn atom in this geometry was present in 498 small-molecule crystal structures (1.87%), further hinting towards a Zn^2+^ at the M1 site. In conclusion, the M1 metal-binding site of FBPases is structurally versatile and can accommodate several cations in different coordination geometries. No metal ions are present at sites M2 and M3 in hlFBPase. The metal ion at site M3 is coordinated by an aspartate side chain from the catalytic loop (Asp68 in Fig. 8 ▸ *b*). Residues 55–70 of the catalytic loop are disordered in the hlFBPase structure, possibly explaining the absence of the other cations.

The allosteric AMP site in hlFBPase is also occupied by a sulfate ion, which mimics the position of the phosphoryl group of AMP (Fig. 8 ▸ *d*). The anion is cradled in a classical P-loop followed by an α-helix. A conserved set of hydrogen bonds from the side chain of a threonine and the NH groups of main-chain residues in the P-loop, together with the positive end of the helix dipole and a lysine side chain, neutralize the negative charges of the anion (Figs. 8 ▸ *e* and 8 ▸ *f*).

## 4. Conclusions   

While a model for the T state of hlFBPase is sufficient for structure-based drug-design purposes, the conformational picture of human liver FBPase has hitherto been incomplete. The structure of hlFBPase in the R state fills this gap. It shows that the R state of hlFBPase is similar to the R states of the rabbit, porcine and human muscle FBPases and that the human liver isoform engages in conformational changes similar in magnitude to those of porcine FBPase. The hlFBPase structure exhibits a number of interesting properties, including a metal ion in a comparatively rare trigonal bipyramidal coordination bound *via* a water molecule to a sulfate ion that mimics the leaving inorganic phosphate after hydrolysis of the substrate. From a crystallographic point of view, the hlFBPase structure is interesting for its peculiar arrangement of NCS elements, which emulate *I*-centred symmetry while the true symmetry is primitive with a *c* axis that is three times longer. A search in the PDB for structures with more than two molecules per asymmetric unit in the four space groups *P*4_1_22, *P*4_3_22, *P*4_1_2_1_2 and *P*4_3_2_1_2 that exhibit pseudo-translation returned 86 instances with a vector >20% of the Patterson origin peak. Of these, 33 emulate *I*-centring with a peak at (1/2, 1/2, *w*), but only three structures, PDB entries 4gqc, 2g6z and 3gfb, have *w* at rational fractions of the *c* axis (*w* = 1/2, *w* = 1/6 and *w* = 1/4, respectively). In none of these cases is the rNCS axis at a position to emulate crystallographic symmetry, so the case of hlFBPase seems unique at present. However, given the high prevalence of pseudo-symmetry in macromolecular crystal structures of ∼8% (Zwart, Grosse-Kunstleve *et al.*, 2008 ▸), similar cases are to be expected.

## Supplementary Material

PDB reference: human liver FBPase, 5ldz

## Figures and Tables

**Table 1:** Data-collection statistics for human liver FBPase

Resolution range† (Å)	39.8–2.2 (2.3–2.2)
100% criterion‡ (Å)	2.2
Patterson symmetry	*P*4/*mmm*
Unit-cell parameters (Å)	*a* = 121.5, *c* = 316.6
Total reflections	910459 (51884)
Unique reflections	121210 (11693)
Multiplicity	7.5 (4.4)
Completeness (%)	99.7 (97.9)
*R*_merge_ §	0.132 (1.22)
*R*_meas_ §	0.142 (1.47)
CC_1/2_ §	0.997 (0.807)
CC*§	0.999 (0.945)
Average *I*/σ(*I*)	7.0 (1.1)
Wilson *B* value (Å^2^)	37.3
Mean |*E* ^2^ − 1|¶	1.152 (0.736/0.541)
〈*I* ^2^〉/〈*I*〉^2^	4.224 (2.0/1.5)
〈*F*〉^2^/〈*F* ^2^〉	0.556 (0.785/0.541)
Mean |*L*|	0.478 (0.5/0.375)
Mean *L* ^2^	0.31 (0.333/0.200)

† With the exception of *E* values, moments and *L* values, values in parentheses are for the highest resolution shell.

‡ The 100% criterion was calculated using *SFTOOLS* from *CCP*4 (Winn *et al.*, 2011 ▸) and represents the resolution in Å of a 100% complete hypothetical data set with the same number of reflections as the measured data.

§ *R* values and CC_1/2_ are defined in Diederichs & Karplus (1997 ▸) and Karplus & Diederichs (2012 ▸), respectively, and were calculated with *PHENIX* (Zwart, Afonine *et al.*, 2008 ▸). *R*_merge_ for the low-resolution shell (39.8–6.1 Å) is 6.0%, indicating that *P*422 is the correct symmetry.

¶ *E* values, moments and *L* values are calculated using *PHENIX* (Zwart, Afonine *et al.*, 2008 ▸) for acentric reflections in the resolution range 10–3.5 Å. Values in parentheses are the expected values for untwinned and perfectly twinned data, respectively.

**Table 2:** Systematic absences

Type	〈*I*〉/〈σ(*I*)〉	No. of reflections
Screw axes		
0*k*0, *k* = 2*n*	16.7	26
0*k*0, *k* = 2*n* + 1	0.9	26
00*l*, *l* = 4*n*	12.2	31
00*l*, *l* ≠ 4*n*	1.1	94
*I*-centring		
*h* + *k* + *l* = 2*n*	12.8	60612
*h* + *k* + *l* = 2*n* + 1	10.5	60598
Pseudo-translation at (1/2, 1/2, 1/6)		
*h* + *k* + *l*/3 = 2*n*	19.6	20386
*h* + *k* + *l*/3 = 2*n* + 1	2.0	20406
Rest	9.7	80418
All	11.8	121210

**Table 3:** Molecular-replacement and refinement statistics in different space groups Molecular replacement was performed with *Phaser* (McCoy *et al.*, 2007 ▸) using a dimer as the search model. Refinement was performed in *BUSTER* (Blanc *et al.*, 2004 ▸).

Space group	RF *Z*-score†	TF *Z*-score‡	Packing violations	LLG§	*R*/*R*_free_ (%)
*P*4_1_22	57.9	97.3	0	27700	0.22/0.25
*P*4_1_2_1_2	57.9	114.5	0	32000	0.21/0.24
*P*4_3_22	52.2	78.7	0	18400	0.25/0.28
*P*4_3_2_1_2	52.2	65.7	1	16800	0.24/0.28

† RF, rotation function.

‡ TF, translation function.

§ LLG, log-likelihood gain.

**Table 4:** Refinement statistics in space group *P*4_1_2_1_2 Values in parentheses are for the highest resolution shell.

PDB code	5ldz
Resolution range (Å)	39.8–2.2 (2.26–2.20)
No. of reflections	120699 (8818)
*R*_cryst_ †	0.206 (0.271)
*R*_free_ †	0.239 (0.286)
DPI‡ (Å)	0.24
Phase error[Table-fn tfn10] (°)	31
R.m.s.d., bonds (Å)	0.012
R.m.s.d., angles (°)	1.66
Ramachandran plot[Table-fn tfn11] (%)	
Favoured	97
Allowed	3
Disallowed	0
*MolProbity* score[Table-fn tfn12]	1.24
Clashscore[Table-fn tfn13]	1.33
No. of protein residues	1872
No. of sulfate ions	29
No. of Zn^2+^ ions	9
No. of Cl^−^ ions	2
No. of atoms	
Protein	14528
Ligands	154
H_2_O	488
〈*B*〉 (Å^2^)	
Overall	46.6
Protein	46.2
Ligands	92.0
H_2_O	43.3

† *R*_cryst_ = 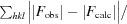 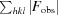, where *F*_obs_ and *F*_calc_ are the structure-factor amplitudes from the data and the model, respectively. *R*_free_ is as *R*_cryst_ but calculated using a 5% test set of structure factors.

‡ Cruickshank diffraction-component precision index based on the *R* value (Blow, 2002 ▸).

§ The maximum-likelihood-based phase error was calculated with *PHENIX* (Zwart, Afonine *et al.*, 2008 ▸).

¶ Calculated using *PHENIX* (Zwart, Afonine *et al.*, 2008 ▸).

†† The *MolProbity* score should approach the high-resolution limit (Chen *et al.*, 2010 ▸).

‡‡ Clashscore is defined as the number of unfavourable all-atom steric overlaps ≥0.4 Å per 1000 atoms (Word *et al.*, 1999 ▸).

**Table 5:** Geometric relations of the six protomers in the asymmetric unit Values in the upper triangle give the translation vectors in fractional coordinates. Note how close some of the translation components are to the rational numbers 1/2 or multiples of 1/3. The lower triangle of data denotes the r.m.s.d. values in Å after the superposition of all main-chain atoms. See Fig. 3 ▸(*a*) for the correspondence of the letters *A*–*F* to the protomers.

	*A*	*B*	*C*	*D*	*E*	*F*
*A*	—	0.507/0.514/0	0/−0.033/−0.331	0.505/0.483/−0.334	0.007/0.005/−0.668	0.514/0.517/−0.664
*B*	0.367	—	0.510/0.477/−0.334	0.002/−0.036/−0.331	0.513/0.522/−0.664	0.003/0.006/−0.668
*C*	0.370	0.182	—	0.508/0.473/−0.003	−0.002/0.016/−0.337	0.506/0.487/−0.333
*D*	0.208	0.353	0.378	—	0.508/0.487/−0.333	−0.009/0.022/−0.337
*E*	0.391	0.222	0.271	0.447	—	0.489/0.482/0.004
*F*	0.280	0.363	0.350	0.273	0.418	—
